# 729. Lassa Fever Associated Hearing Loss

**DOI:** 10.1093/ofid/ofab466.926

**Published:** 2021-12-04

**Authors:** Samuel Ficenec, Donald Grant, Susan Emmett, John Schieffelin

**Affiliations:** 1 Tulane School of Medicine, New Orleans, Louisiana; 2 Kenema Governement Hospital, Kenema, Eastern, Sierra Leone; 3 Duke University, Durham, North Carolina; 4 Tulane University, New Orleans, Louisiana

## Abstract

**Background:**

Hearing loss (HL) is the second leading cause of disability affecting approximately 19% of the world’s population. Despite well known social, economic, and neurologic consequences this condition receives little attention. Lassa Fever (LF) was noted to be associated with HL shortly after its discovery in the 1970’s. However, the true burden of this sequelae is likely underestimated due to a lack of standardized measurement and reporting.

**Methods:**

We performed a cross-sectional study of LF survivors and household controls in Kenema, Sierra Leone. Upon recruitment, survivors and controls were screened for HL by determining Pure Tone Averages (PTA) of air conduction thresholds using an AMBCO audiometer, according to WHO standards. Individuals found to have elevated PTAs were referred to confirmatory testing measuring both air and bone thresholds using a SHOEBOX audiometer to differentiate sensorineural and conductive HL. All subjects completed symptom questionnaires and physical exams to understand the full spectrum of viral sequelae.

**Results:**

94 LF survivors and 281 controls were recruited. The average age of LF survivors was higher than controls (32.9 vs 28.7, p=0.008). Of these 94 LF survivors, 40 (43%) were found to have HL in comparison to 40 (14%) of controls (p< 0.001). Lassa fever survivors were also found to have significantly worse HL with 16 (40%) found to have profound HL compared to only 2 (5%) of controls (p< 0.001). Logistic regression of this cohort found that LF infection (OR = 1.30, p< 0.001), any inner or middle ear symptoms (OR = 1.20, p=0.041), or pharyngeal symptoms (OR = 1.23, p=0.012) were significant risk factors of developing HL (p< 0.001). Interestingly the development of any pulmonary symptoms was protective of HL (OR = 0.86, p=0.039). Animal model studies suggested that LF infection may result in the development of an ANCA vasculitis which may be causative of LF sequelae. A subset of LF survivors (n=80) and IgG negative controls (n=9) were tested for ANCA proteins, of these 20 (25%) survivors vs 5 (55%) tested positive with mean concentrations of 202.4 µg/ml and 135.7 µg/ml (p=0.449), respectively.

**Conclusion:**

This data further characterizes the sequelae of LF and suggests mechanisms of pathogenesis of symptoms.

**Disclosures:**

**All Authors**: No reported disclosures

